# Laparoendoscopic single-site nephrectomy versus conventional laparoendoscopic nephrectomy for kidney tumor: a systematic review and meta-analysis

**DOI:** 10.1042/BSR20190014

**Published:** 2019-08-09

**Authors:** Dengyuan Feng, Rong Cong, Hong Cheng, Yi Wang, Jiajun Zhou, Jiadong Xia, Min Gu

**Affiliations:** 1 Department of Urology, The First Affiliated Hospital of Nanjing Medical University, Nanjing 210008, China; 2 Department of Urology, Zhongda Hospital Affiliated to Southestern China University, Nanjing 210009, China; 3 Department of Urology, the Second Affiliated Hospital of Nanjing Medical University, Nanjing 210011, China

**Keywords:** CL-N, conventional laparoendoscopic nephrectomy, kidney tumor, laparoendoscopic single-site nephrectomy, LESS-N, meta-analysis

## Abstract

With the increasing application of laparoendoscopic single-site nephrectomy (LESS-N) in kidney tumor, accumulating studies compared it with conventional laparoendoscopic nephrectomy (CL-N). However, controversial outcomes were reported. Hence, this meta-analysis was carried out to clarify these issues. Online databases PubMed, EMBASE and the Cochrane Library were searched comprehensively for eligible studies published before 24 July 2018. Odds ratios (ORs) or standardized mean differences (SMDs) with corresponding 95% confidence intervals (CIs) were collected for evaluating the pooled results of relevant outcomes. Ultimately, 13 eligible articles were enrolled. Meanwhile, compared with CL-N, LESS-N was related to a longer operation time (SMD: 0.40; 95% CI, 0.23–0.58; *P*=0.000), a shorter length of hospital stay (LOS) (SMD: −0.32; 95% CI, −0.62 to −0.02; *P*=0.034), a lower visual analog scale (VAS) score (SMD: −0.89; 95% CI, −1.22 to −0.56; *P*=0.000) and a lower analgesic requirement (SMD: −0.55; 95% CI, −0.87 to −0.23; *P*=0.001). There was no statistical difference in the postoperative day of oral intake, estimated blood loss (EBL), conversion rate, perioperative complications, intraoperative complications, postoperative complications, minor complications and major complications between LESS-N and CL-N. Patients with LESS-N for kidney tumor could have a longer operation time and shorter LOS, and meanwhile could need less analgesics and suffer less pain after LESS-N.

## Introduction

Over the last decades, open nephrectomy for kidney tumor has been gradually replaced by laparoendoscopic nephrectomy first reported by Clayman et al. in 1991 [1] and it has been demonstrated to gain great advantages in cosmetic appearance, postoperative pain, hospital stay and perioperative complications [2,3]. Recently, new less invasive technologies have been applied in treatment including Natural orifice transluminal endoscopic surgery (NOTES) [4] and laparoendoscopic single-site (LESS) surgery [5]. However, NOTES needs special equipment and more technical skills, which limits its application [4]. As a result, LESS has gained a lot of attention since it was first reported in *Urology* in 2007 [5].

As for LESS nephrectomy (LESS-N), it is a kind of minimally invasive surgery based on LESS, which is performed through a single incision, usually around the umbilical or transperitoneal region. Sometimes, an additional 3-mm trocar used in right kidney nephrectomies for liver retraction is also considered as LESS-N [5,6]. In terms of conventional laparoendoscopic nephrectomy (CL-N), it is also a minimally invasive surgery accomplished through usually three keyholes. Nowadays, more and more researches focus on the comparison of LESS-N and CL-N, including operation time, estimated blood loss (EBL), postoperative day of oral intake, length of hospital stay (LOS), visual analog scale score (VAS), analgesic requirement, conversion rates, perioperative complications, intraoperative complications, postoperative complications, minor complication and major complications. However, most of them are small series, even with conflicting results.

As a powerful tool, meta-analysis could provide more reliable results than a single study by combining all eligible studies, especially in explaining controversial conclusions. Therefore, we systematically and comprehensively searched eligible articles and evaluated their potential efficiency, safety and advantages of LESS-N in comparison of CL-N.

## Materials and methods

### Literature search

PubMed, EMBASE and the Cochrane Library were searched comprehensively for eligible studies published before 24 July 2018. The search strategy consisted of the following keywords in combination with Medical Subject Headings (MeSH) terms and text words: (‘single site laparoscopy/laparoendoscopy’ or ‘single port laparoscopy/laparoendoscopy’) and (‘conventional laparoscopy/laparoendoscopy’ or ‘traditional laparoscopy/laparoendoscopy’) and ‘nephrectomy’. Meanwhile, additional articles were searched in the database manually, when we searched relevant reviews and the reference list of original articles.

### Inclusion and exclusion criteria

The eligible studies needed to meet the following inclusion criteria: (1) studies comparing LESS-N with CL-N for kidney tumor; (2) reporting at least one of the following perioperative outcomes: operation time, EBL, postoperative day of oral intake, LOS, VAS, analgesic requirement, conversion rate, perioperative complications, intraoperative complications, postoperative complications, minor complication or major complications; (3) sufficient data could be extracted from the enrolled studies. Additionally, the exclusion criteria included the following points: (1) not meet the inclusion criteria; (2) reviews, conference meeting abstracts, case reports or comments; (3) data could not be extracted; (4) overlapping data from the same institution.

### Data extraction and study quality

The preferred reporting items for systematic review and meta-analysis (PRISMA) was utilized for reporting this article [7]. Two independent authors were responsible for extracting data from the included studies. Demographic characteristics were compared including age, gender ratio, body mass index (BMI), side of procedure, tumor size, American Society of Anesthesiologists score (ASA), history of prior abdominal surgery. The perioperative outcomes were collected including operation time, EBL, postoperative day of oral intake, LOS, VAS, analgesic requirement, conversion rate, perioperative complications, intraoperative complications, postoperative complications, minor complication and major complications. Postoperative complications were graded according to the Clavien-Dindo system [8]. The evidence level of included studies was rated by one author according to the criteria provided by the Oxford Centre for Evidence-Based Medicine. The methodological quality of randomized controlled trial (RCT) was evaluated by the Jadad scale [9]. Meanwhile, the methodological quality of the retrospective studies was assessed by the modified Newcastle–Ottawa scale [10].

### Statistical analysis

Stata 14.0 was utilized to perform this meta-analysis. All statistical methods met the principles mentioned in PRISMA. The standardized mean differences (SMDs) or odds ratios (ORs) were used for continuous and dichotomous variables, respectively; 95% confidence intervals (CIs) were reported in all results. Studies presenting continuous data as means and range values, the standard deviations were calculated using the methods described by Hozo et al. [11]. Statistical heterogeneity was evaluated by chi-square test and inconsistency (*I^2^*). If high heterogeneity was tested, a random-effect model was used. Otherwise, a fixed-effect model would be applied. The pooled effects were tested by the Z-test and the results with *P*<0.05 were considered to be statistically significant. Contour-enhanced funnel plots and L’abbe graph were used to evaluate publication bias for dichotomous variables and continuous variables, respectively.

## Results

### Characteristics and quality of included studies

**Figure 1:**
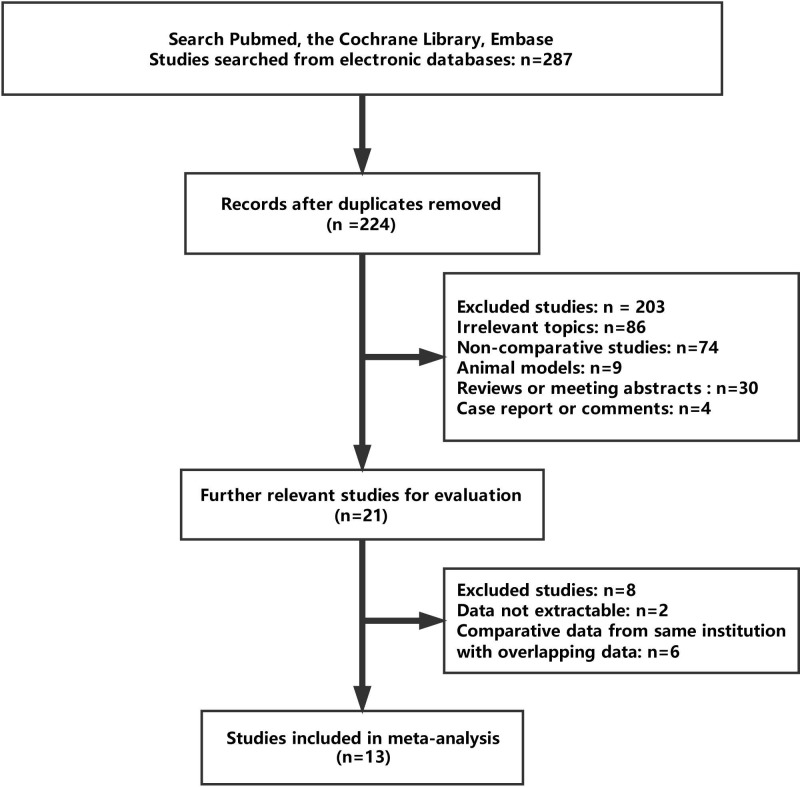
Flow chart of the study selection process

A total of 13 eligible studies were ultimately enrolled in this meta-analysis with 822 patients undergoing LESS-N (*n*=336, 40.9%) and CL-N (*n*=486, 59.1%). Characteristics and results of all these studies are displayed in Tables 1 and 2, respectively. Moreover, the studies selection process is presented in Figure 1. Therein, one study was a small sample RCT (level of evidence: 2b) [12]. Eight studies used contemporaneous patients as control groups (level of evidence: 3b) [13–21], and one of those studies reported collecting data prospectively [14]. Three retrospective studies used historical patients as control groups (level of evidence: 4) [22–24]. Ten studies reported about radical nephrectomy (RN) [12–17,21,22–24]. Two studies were about partial nephrectomy (PN) [19,20]. Both RN and PN were reported by Kim et al. [18], so we used Kim (RN) and Kim (PN) as RN and PN in the present study, respectively. Two studies reported robot-assisted nephrectomy [14,19].

**Table 1:** Characteristics of these enrolled studies in this meta-analysis

Study, year	Level of evidence	Design	Indications	Quality score^1^ (*)	Number of patients		Ports for LESS	Matching
					LESS-N	CL-N		
Park, 2010	4	R	RN	*******	19	38	Homemade/OCTO Port	1,2,3,4,5,6,7
Seo, 2011	3b	R	RN	*******	10	12	Homemade/SILS-Port	1,2,3,4,6,7,8
Zhang, 2011	3b	R	RN	*******	10	15	Homemade	1,2,3,4,7,8
White, 2011	3b	RP	RN	*******	10	10	SILS Port/GelPort	1,2,3,4,5,7,8
Wang, 2012	3b	R	RN	*******	20	33	TriPort	1,2,3,4,5,7,8
Greco, 2012	4	R	RN	*******	31	35	Endocone	1,2,3,4,5,7,8
Antonelli, 2013	4	R	RN	*******	47	94	No#	1,2,3,5,6,7,8
Dong, 2013	3b	R	RN	******	29	29	Homemade	1,2,3,7
Kim, 2013	3b	R	RN	*******	26	14	Homemade	1,2,3,4,6,8
Kim, 2013	3b	R	PN	*******	5	16	Homemade	1,2,3,4,6,8
Shin, 2014	3b	R	PN	*******	79	80	Homemade	1,2,3,4,5,7
Park, 2015	2b	RCT	RN	***,^2^	17	18	Octoport	1,2,3,4,5,6,7,8
Wolters, 2015	3b	R	PN	*******	13	72	No#	1,2,3,4,5,6,7
Feng, 2016	3b	R	RN	*******	20	20	No#	1,2,3,4,7,8

Abbreviations: R, retrospective; RP, retrospective design, prospective data collection collection. Matching: 1 = age, 2 = gender ratio, 3 = BMI, 4 = side of procedure, 5 = ASA, 6 = history of prior abdominal surgery, 7 = tumor size, 8 = same surgeon. ^1^No single-port access device was used; the adjacent trocars were inserted through a single incision. ^2^The score of the Jadad scale for the methodological quality of the RCTs.

**Table 2:** Results of meta-analysis comparison of LESS-N and CL-N two groups and LESS-RN and CL-RN two groups

Perioperative outcomes	Studies, number	LESS-N/LESS-RN patients, number	CL-N/CL-RN patients, number	WMD/OR (95% CI)	*P*-value	Study heterogeneity			
						X^2^	df	*I^2^*, %	*P*-value
**Perioperative outcomes for LESS-N and CL-N**									
Operation time, min	9	233	335	0.404 (0.226, 0.581)	0.000	17.53	9	48.7	0.041
EBL, ml	7	210	248	−0.214 (−0.516, 0.088)	0.165	15.06	7	53.5	0.035
Postoperative day of oral intake, d	5	100	141	−0.256 (−0.847, 0.335)	0.397	4.83	2	58.6	0.089
Length of stay, d	9	252	352	−0.319 (−0.615, −0.023)	0.034	23.90	9	62.3	0.004
VAS	7	210	251	−0.892 (−1.223, −0.561)	0.000	16.84	7	58.4	0.018
Analgesic requirement, mg	3	70	93	−0.550 (−0.870, −0.229)	0.001	2.31	2	13.5	0.315
Conversion rate	9	235	377	^1^1.685 (0.617, 4.606)	0.309	4.22	4	5.2	0.377
Perioperative complications	8	156	195	^1^1.742 (0.932, 3.254)	0.082	9.39	8	14.8	0.310
Intraoperative complications	5	176	284	^1^1.537 (0.585, 4.036)	0.383	1.42	3	0.0	0.701
Postoperative complications	6	186	294	^1^1.353 (0.817, 2.241)	0.240	1.55	5	0.0	0.907
Minor complications	5	166	274	^1^1.250 (0.722, 2.163)	0.426	0.26	4	0.0	0.992
Major complications	5	166	274	^1^2.132 (0.587, 7.740)	0.250	1.45	2	0.0	0.483
**Perioperative outcomes for LESS-RN and CL-RN**									
Operation time, min	7	136	167	0.527 (0.120, 0.934)	0.011	16.58	6	63.8	0.011
EBL, ml	6	126	152	−0.234 (−0.594, 0.127)	0.204	10.46	5	52.2	0.063
Postoperative day of oral intake, d	5	100	141	−0.256 (−0.847, 0.335)	0.397	4.83	2	58.6	0.089
Length of stay, d	7	155	184	−0.390 (−0.759, −0.020)	0.039	16.12	6	62.8	0.013
VAS	6	126	155	−1.065 (−1.322, −0.808)	0.000	4.53	5	0.0	0.475
Analgesic requirement, mg	3	70	93	−0.550 (−0.870, −0.229)	0.001	2.31	2	13.5	0.315
Conversion rate	7	143	225	^1^4.209 (0.916, 19.346)	0.065	1.91	2	0.0	0.385
Perioperative complications	8	151	179	^1^1.412 (0.728, 2.740)	0.308	4.76	7	0.0	0.690
Intraoperative complications	3	84	132	^1^1.602 (0.364, 7.037)	0.533	1.34	1	25.4	0.247
Postoperative complications	4	94	142	^1^1.408 (0.598, 3.316)	0.434	1.06	3	0.0	0.788

Abbreviations: CL-RN, conventional laparoscopic RN; df, degree of freedom; LESS-RN, laparoscopic single-site RN; WMD/OR, weighted mean difference/odds ratio. ^1^Odds ratio.

### LESS-N compared with CL-N

**Figure 2:**
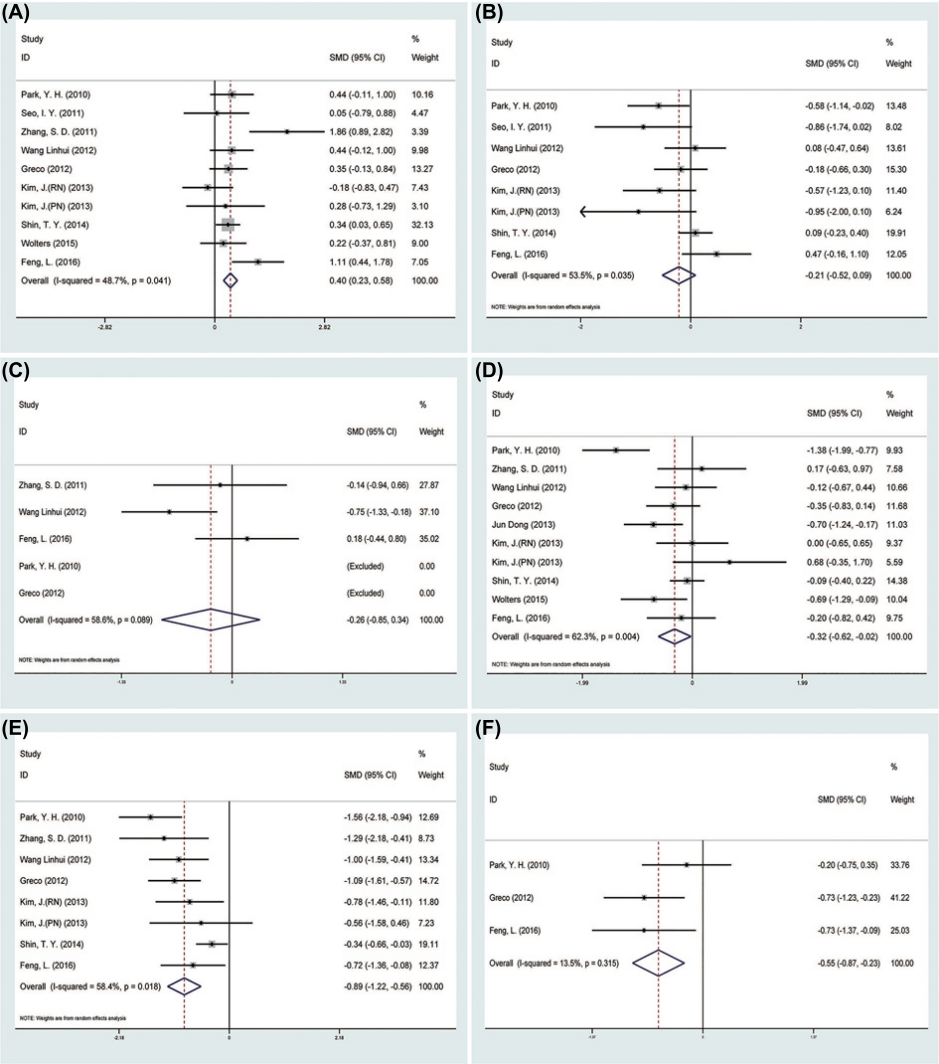
Forest plots of the outcomes between LESS-N and CL-N (**A**) Indicated the SMD of operation time; (**B**) indicated the SMD of EBL; (**C**) indicated the SMD of postoperative day of oral intake; (**D**) indicated the SMD of LOS; (**E**) indicated the SMD of VAS; (**F**) indicated the SMD of analgesic requirement.

A total of nine studies [13,15,16,18–23] reported operation time for the 568 patients, which is longer in the LESS-N group than the CL-N group (SMD = 0.40 min, 95% CI = 0.23–0.58; *P*=0.000) (Figure 2A). As for EBL, pooled data from seven studies [13,16,18–19,21–23] reported that there was no difference in between the CL-N group and the LESS-N group (SMD = −0.21 ml, 95% CI = −0.52 to 0.09; *P*=0.165) (Figure 2B), so was the postoperative day of oral intake (SMD = −0.26 d, 95% CI = −0.85 to 0.34; *P*=0.397) [15–16,21,22–23] (Figure 2C). Nine studies [15–23] reported LOS in 604 patients, and the pooled data favored the LESS-N group (SMD = −0.32 d, 95% CI = −0.62 to −0.02; *P*=0.034) (Figure 2D). Postoperative pain was evaluated by means of VAS in seven studies [15–16,18–19,21–23] including 461 patients. The pooled data showed lower VAS score in the LESS-N group than CL-N group (SMD = −0.89, 95% CI = −1.22 to −0.56; *P*=0.000) (Figure 2E). Three studies [21–23] reported analgesic requirement and the pooled data showed the LESS-N group had lower analgesic requirement than the CL-N group (SMD = −0.55 mg, 95% CI = −0.87 to −0.23; *P*=0.001) (Figure 2F).

**Figure 3:**
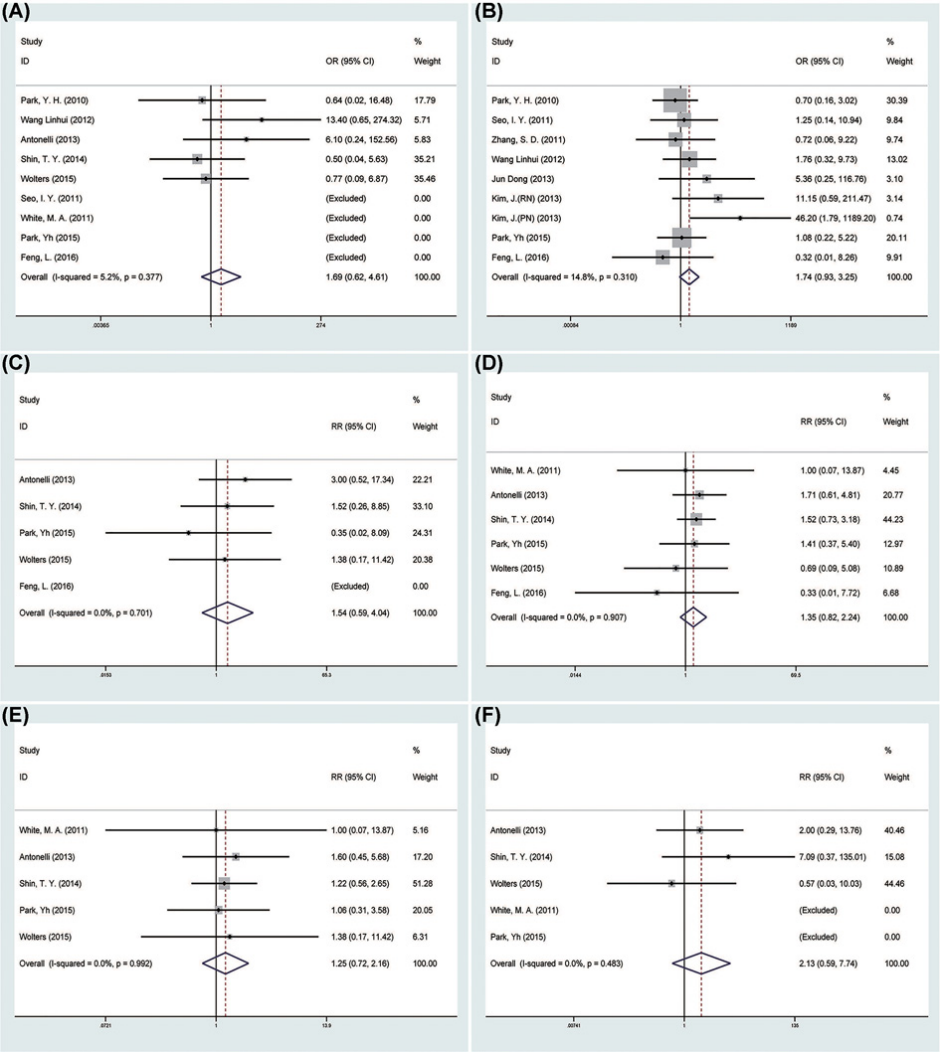
Forest plots of the outcomes between LESS-N and CL-N (**A**) Indicated the OR of conversion rate; (**B**) indicated the OR of perioperative complications; (**C**) indicated the OR of intraoperative complications; (**D**) indicated the OR of postoperative complications; (**E**) indicated the OR of minor complications; (**F**) indicated the OR of major complications.

There was no statistical difference in the conversion rate between the two groups (OR = 1.69, 95% CI = 0.62–4.61; *P*=0.309) [12–14,16,19–22,24] (Figure 3A). Eight studies [12–13,15–18,21–22] reported perioperative complications in 351 patients, and the pooled data showed no difference between the LESS-N group and the CL-N group (OR = 1.74, 95% CI = 0.93–3.25; *P*=0.082) (Figure 3B). The pooled data of intraoperative complications [12,19–21,24] and postoperative complications [12,13,19–21,24], both showed no statistically difference between the two groups (OR = 1.54, 95%CI = 0.59–4.04, *P*=0.383; OR = 1.35, 95% CI = 0.82–2.24, *P*=0.240, respectively) (Figure 3C,D). Five studies [12,14,19–20,24] divided postoperative complications into minor and major complications and the pooled data both showed no difference in minor complications and major complications between two groups (OR = 1.25, 95% CI = 0.72–2.16, *P*=0.426; OR = 2.13, 95% CI = 0.59–7.74, *P*=0.250) (Figure 3E,F).

### Laparoendoscopic single-site nephrectomy compared with conventional laparoscopic RN

**Figure 4:**
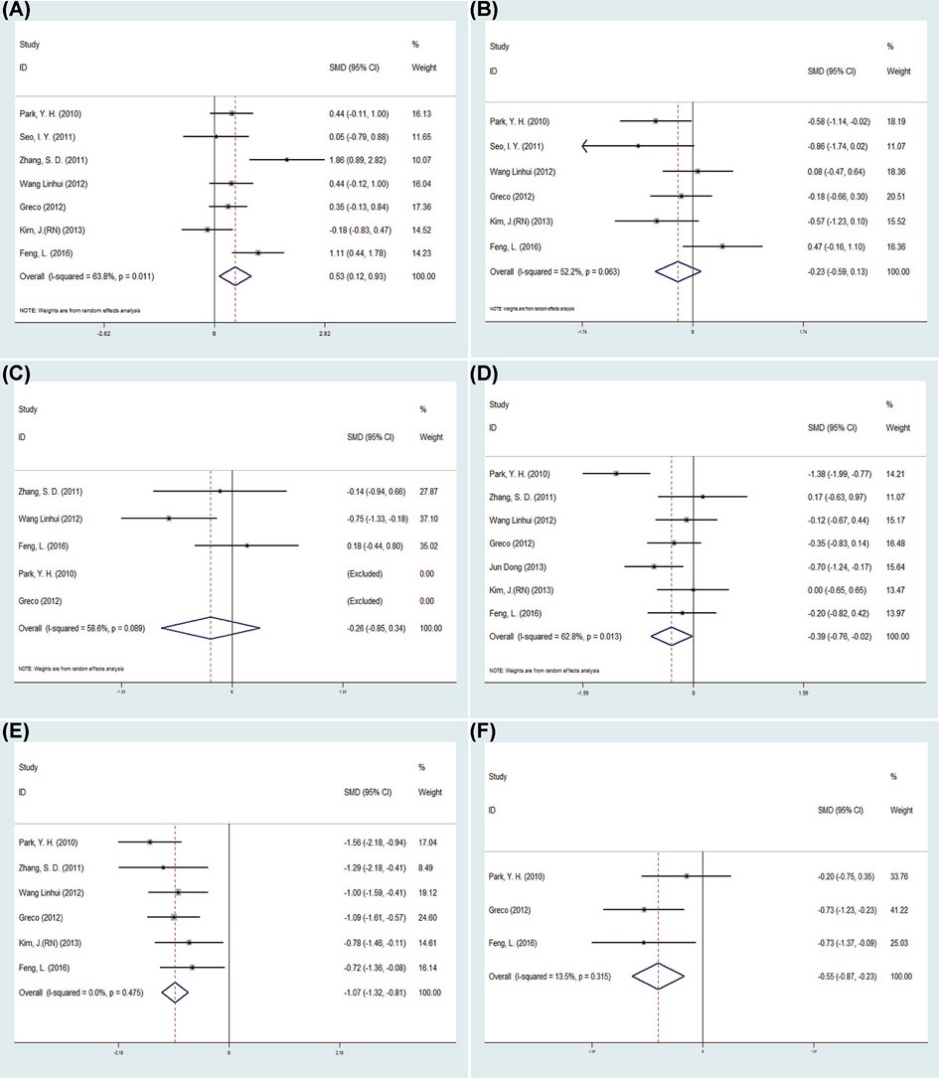
Forest plots of the outcomes between LESS-RN and CL-RN (**A**) Indicated the SMD of operation time; (**B**) indicated the SMD of operation time EBL; (**C**) indicated the SMD of postoperative day of oral intake; (**D**) indicated the SMD of LOS; (**E**) indicated the SMD of VAS; (**F**) indicated the SMD of analgesic requirement.

The pooled data about postoperative day of oral intake and analgesic requirement between the LESS-N and CL-N two groups and the laparoendoscopic single-site RN (LESS-RN) and conventional laparoscopic RN (CL-RN) two groups were exactly same and so were the results (Figure 4C–F). The pooled data showed that the LESS-RN group had longer operation time than the CL-RN group (SMD = 0.53 min, 95% CI = 0.12–0.93; *P*=0.011) [13,15–16,18,21–23] (Figure 4A). Moreover, there was no statistical difference in the EBL between the two groups (SMD = −0.23 ml, 95% CI = −0.59 to 0.13; *P*=0.204) [13,16,18,21–23] (Figure 4B). Seven studies [15–18,21–23] reported the LOS in 339 patients and the pooled data favored the LESS-RN group (SMD = −0.39d, 95% CI = −0.76 to −0.02, *P*=0.039) (Figure 4D). Six studies [15–16,18,21–23] evaluated postoperative pain using VAS score, and the LESS-RN had a lower score than the CL-RN group statistically (SMD = −1.07, 95% CI = −1.32 to −0.81, *P*=0.000) (Figure 4E).

**Figure 5:**
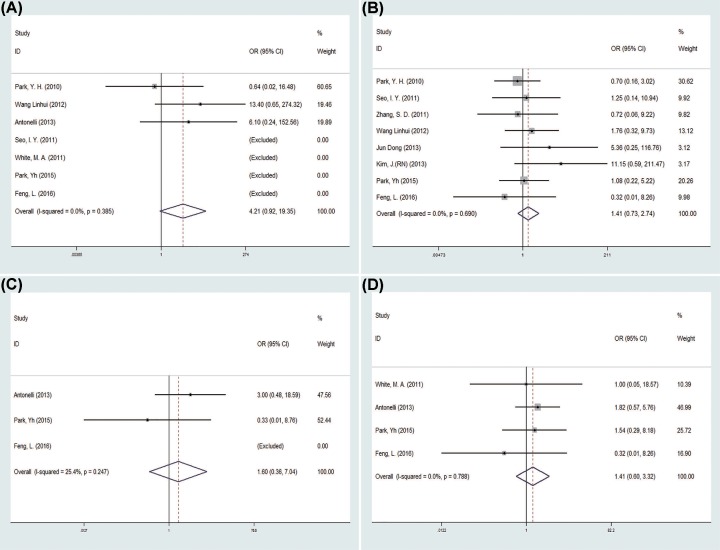
Forest plots of the outcomes between LESS-RN and CL-RN (**A**) Indicated the OR of conversion rate; (**B**) indicated the OR of perioperative complications; (**C**) indicated the OR of intraoperative complications; (**D**) indicated the OR of postoperative complications.

The LESS-RN group had higher conversion rate than the CL-RN group, but the pooled data show no statistical difference between two groups (OR = 4.21, 95% CI = 0.92–19.35; *P*=0.065) [12–14,16,21–22,24] (Figure 5A). Eight studies [12–13,15–18,21–22] reported perioperative complications in 330 patients. There was no difference between two groups (OR = 1.41, 95% CI = 0.73–2.74, *P*=0.308) (Figure 5B). Both intraoperative complications [12,21,24] and postoperative complications [12,14,21,24] showed no difference between the two groups (OR = 1.60; 95% CI = 0.36–7.04, *P*=0.247; OR = 1.41, 95% CI = 0.59–3.32, *P*=0.434, separately) (Figure 5C,D). Most of postoperative complications were minor complications; 81.8% in the LESS-RN group and 83.3% in the CL-RN group.

### Publication bias

**Figure 6:**
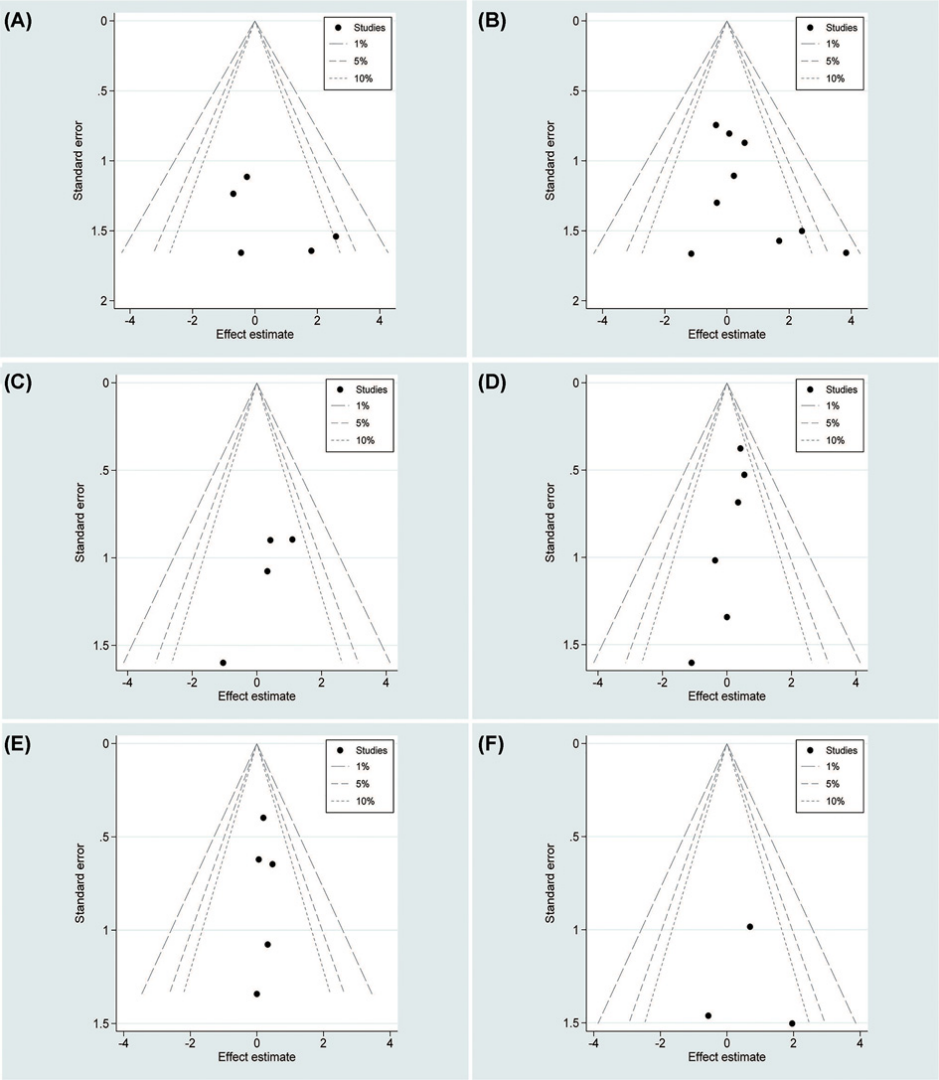
Contour-enhanced funnel plots for count variables for LESS-N and CL-N two groups (**A**) Conversion rate; (**B**) perioperative complications; (**C**) intraoperative complications; (**D**) postoperative complications; (**E**) minor complications; (**F**) major complications.

**Figure 7:**
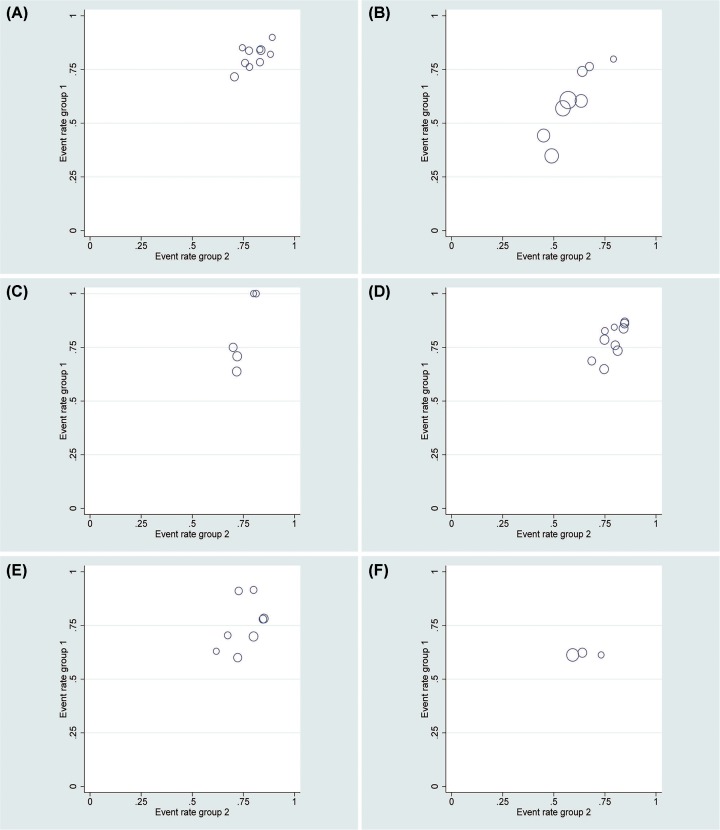
L’Abbe graphs for continuous variables for LESS-N and CL-N two groups (**A**) Operation time; (**B**) EBL; (**C**) postoperative day of oral intake; (**D**) LOS; (**E**) VAS; (**F**) analgesic requirement.

**Figure 8:**
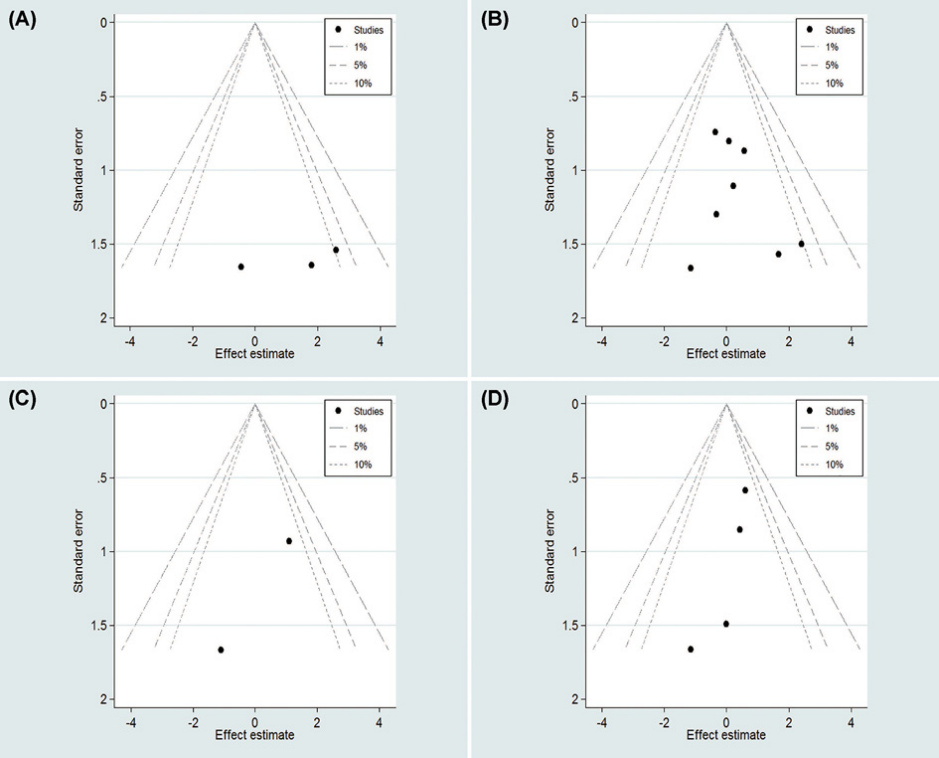
Contour-enhanced funnel plots for count variables for LESS-RN and CL-RN two groups (**A**) Conversion rate; (**B**) perioperative complications; (**C**) intraoperative complications; (**D**) postoperative complications.

**Figure 9:**
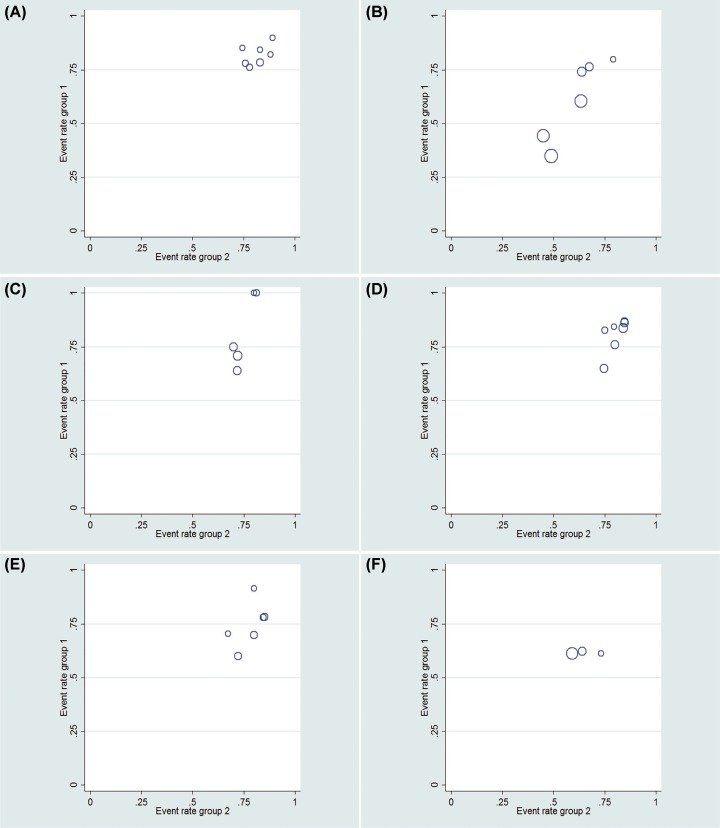
L’Abbe graphs for continuous variables for LESS-RN and CL-RN two groups (**A**) Operation time; (**B**) EBL; (**C**) postoperative day of oral intake; (**D**) LOS; (**E**) VAS; (**F**) analgesic requirement.

In this meta-analysis, contour-enhanced funnel plots were used for dichotomous variables (Figures 6 and 8). L’Abbe graphs were used for continuous variables (Figures 7 and 9). No publication bias was found in our study.

## Discussion

This meta-analysis compared not only LESS-N with CL-N, but also LESS-RN with CL-RN for kidney tumor and the pooled results analyzed in this article, showed that LESS-N was related to a longer operation time, a shorter LOS, a lower VAS and a lower analgesic requirement. Moreover, LESS-N had similar perioperative outcomes with CL-N including postoperative day of oral intake, EBL, conversion rate, perioperative complications, intraoperative complications, postoperative complications, minor complications and major complications. As for the analysis of LESS-RN and CL-RN, there were no significant differences compared with the analysis of LESS-N and CL-N.

In this article, we found operation time of LESS-N was longer than CL-N. LESS operation is difficult and requires technical proficiency, laparoendoscopic operation skills and high capability of handling emergency. Surgeons contacting LESS-N in the early stage are hard to master this technology quickly [15]. The learning curve of LESS-N is quite long, which could be the main reason for longer operation time for LESS-N. Park et al. [22] reported that operation time decreased with the increasing number of patients. So extensive training in LESS-N will reduce operation time. Meanwhile, proper devices for LESS-N could make the operation easier. Some institutions utilize homemade devices to obtain satisfactory outcomes [13,15,17–19,22,25]. Some others use special devices like OCTO Port, SILS-Port and GelPort etc [12–14,16,22–23].

The safety and feasibility of LESS-N is of great concern. Special devices and related training applied made sure surgeons could perform LESS-N as well as CL-N. Conversion rate was a good indicator for evaluating whether the surgery is safe and feasible. Conversion into open surgery or conventional laparoscopy surgery was inevitable for some situations such as bleeding etc. The pooled data showed conversion rate of LESS-N and CL-N was 2.6 and 3.0%, respectively. Meanwhile no difference in conversion rate was found between two groups. The incidence of complications is widely recognized as an important indicator of surgical safety. The Clavien-Dindo system classified perioperative complications into five grades, in which grades 1 and 2 are considered as minor complications, meanwhile grades 3, 4 and 5 are thought as major complications [8]. Complications after nephrectomy were dominantly minor complications, which included fever, diarrhea, ileus, bleeding etc. Major complications such as hydronephrosis, severe pneumonia, intensive care after surgery rarely happened [19–22]. In this meta-analysis, there is also no significant difference in perioperative complications, intraoperative complications, postoperative complications, major complications and minor complications between LESS-N and CL-N.

Results from this meta-analysis demonstrated lower VAS and analgesic requirement in LESS-N group, which was similar to a previously published meta-analysis [26] showing that patients with LESS-N suffer less postoperative pain than patients with CL-N. LESS-N and CL-N are considered as minimally invasive surgeries, and the main difference between the two surgical procedures is the number of ports applied. Single one incision with less muscle-splitting in LESS-N is considered a main reason [16,21]. In CL-N, removing specimen needs a corresponding incision. However, surgical procedure and removal of specimen are operated through one incision in LESS-N, which also reduces surgical scar and leads to a better cosmetic. Several studies [27–29] reported morcellating specimen may also decrease postoperative discomfort. However, morcellation might induce tumor seeding, which limits its use in surgery [16].

Some researchers studied warm ischemic time, which affects renal function after nephrectomy by influencing blood supply of renal parenchyma. Shin et al. [19] reported LESS-PN had a longer mean warm ischemic time than CL-PN (19.8 ± 13.1 vs. 26.5 ± 10.5 min, *P*=0.001). However, postoperative renal function, evaluated by preoperative to postoperative eGFR change, was only found significantly higher in CL-PN than LESS-PN on postoperative day 1 but there was no difference between CL-PN and LESS-PN on postoperative days 0, 1, 7, and at 3 and 6 months, demonstrating warm ischemic time primarily affected renal function within a short period after nephrectomy. Meanwhile, Springer et al. [30] reported percent of eGFR preserved affected postoperative renal function primarily other than warm ischemic time. So size and complexity of tumor determining percent of parenchyma preserved dominantly indicates renal function postoperatively.

The meta-analysis has some limitations that should not be ignored. The main limitation is that nearly all the studies included were retrospective, except one prospective non-randomized study and one RCT. Besides, most studies are small sample studies. Second, surgeons with different surgical experience used diverse devices. The background of each study was different. But the results of the LESS-RN and CL-RN are the same with the results of the LESS-N and CL-N. In the end, all studies lack long period of follow-up, which means that recurrence, metastasis and mortality could not be evaluated. So long-term follow-up between LESS-N and CL-N is really needed. Nevertheless, this meta-analysis provides latest information on comparing LESS-N and CL-N for kidney tumor. The conclusion is quite convincing because of a large number of patients. We not only analyzed the perioperative outcomes of LESS-N and CL-N, but also the perioperative outcomes of LESS-RN and CL-RN. Different ways to evaluate methodological quality of included studies were applied. And contour-enhanced funnel plots and L’Abbe graphs were applied in this meta-analysis showing no publication bias.

## Conclusions

Taken together, our results shed light on that patients with LESS-N for kidney tumor could have a longer operation time and shorter LOS, and meanwhile could need less analgesics and suffer less pain after LESS-N. Due to the aforementioned limitations, larger samples of more strictly designed RCTs are required to verify our findings.

## Abbreviations

BMI: body mass index
CI: confidence interval
CL-N: conventional laparoendoscopic nephrectomy
CL-RN: conventional laparoscopic radical nephrectomy
EBL: estimated blood loss
LESS-N: laparoendoscopic single-site nephrectomy
LESS-RN: laparoendoscopic single-site radical nephrectomy
LOS: length of hospital stay
NOTES: natural orifice transluminal endoscopic surgery
OR: odds ratio
PN: partial nephrectomy
PRISMA: preferred reporting items for systematic review and meta-analysis
RCT: randomized controlled trial
RN: radical nephrectomy
SMD: standardized mean difference
VAS: visual analog scale score
